# P53 aggregation, interactions with tau, and impaired DNA damage response in Alzheimer’s disease

**DOI:** 10.1186/s40478-020-01012-6

**Published:** 2020-08-10

**Authors:** Kathleen M. Farmer, Gaurav Ghag, Nicha Puangmalai, Mauro Montalbano, Nemil Bhatt, Rakez Kayed

**Affiliations:** 1 Mitchell Center for Neurodegenerative Diseases, University of Texas Medical Branch, 301 University Blvd, Medical Research Building, Room 10.138C, Galveston, TX 77555-1045 USA; 2 Departments of Neurology, Neuroscience and Cell Biology, University of Texas Medical Branch, Galveston, TX USA; 3 Protein Sciences, Merck & Co Incorporated, South San Francisco, CA USA

**Keywords:** p53, Tau, Oligomers, DNA damage, Seeding, Cross-seeding, Alzheimer’s disease

## Abstract

The transcription factor, p53, is critical for many important cellular functions involved in genome integrity, including cell cycle control, DNA damage response, and apoptosis. Disruption of p53 results in a wide range of disorders including cancer, metabolic diseases, and neurodegenerative diseases. Alzheimer’s disease (AD) is a neurodegenerative disorder characterized by protein aggregates that contribute to disease pathology. Although p53 is known to aggregate, its propensity to aggregate in AD has never been assessed. Moreover, AD neuropathology includes lethal cell cycle re-entry, excessive DNA damage, and abnormal cell death which are all controlled by p53. Here, we show p53 forms oligomers and fibrils in human AD brain, but not control brain. p53 oligomers can also be detected in htau and P301L mouse models. Additionally, we demonstrate that p53 interacts with tau, specifically tau oligomers, in AD brain and can be recapitulated by in vitro exogenous tau oligomer treatment in C57BL/6 primary neurons. p53 oligomers also colocalize, potentially seeding, endogenous p53 in primary neurons. Lastly, we demonstrate that in the presence of DNA damage, phosphorylated p53 is mislocalized outside the nucleus and p53-mediated DNA damage responders are significantly decreased in AD brain. Control brain shows a healthy DNA damage response, indicating a loss of nuclear p53 function in AD may be due to p53 aggregation and/or interactions with tau oligomers. Given the critical role of p53 in cellular physiology, the disruption of this crucial transcription factor may set an irreversible course towards neurodegeneration in AD and potentially other tauopathies, warranting further investigation.

## Introduction

Alzheimer’s disease (AD) is a devastating neurodegenerative disorder that causes gradual, progressive, and irreversible neuronal death, culminating in nervous system dysfunction, primarily in the form of memory impairment. There are many known contributing factors involved in AD, making it a complex disease. The gradual cell death seen in AD has been historically attributed to tau and amyloid-β protein aggregation, forming the hallmark neurofibrillary tangles (NFTs) and plaques, respectively, seen in AD [41, 60]. However, recent studies suggest that other essential proteins form aggregates that may interact and contribute to toxicity [11, 43, 86, 110].

The transcription factor, p53 is a powerful surveyor of cellular stress and orchestrates a coordinated response to cellular insults [30, 45, 61]. Widely recognized for its action as a tumor suppressor [33, 56, 62, 114], p53 is a much more dynamic protein, acting as a signaling hub [51] for many critical cellular processes such as apoptosis [61, 122], DNA damage repair [52], and cell cycle control [52], heeding its name “guardian of the genome” [61] and “the emergency brake” [58]. Additionally, p53 is an intrinsically disordered protein, making it prone to aggregation [50, 112]. Several studies, including previous reports from our research group, have demonstrated that recombinant p53, both domain fragments and full-length, can spontaneously aggregate and form fibrillar aggregates in vitro [44, 47, 69, 96]. Moreover, both our laboratory and others have demonstrated that this aggregation can cause loss of function and/or gain of function in vitro [4, 38, 66, 96, 120]*.* Additionally, studies have demonstrated that exogenous p53 aggregates can seed and cross-seed [4, 13, 34, 38, 66, 93, 120] and spread in a prion-like manner [4, 13, 34, 93]. To date, these findings have been demonstrated in cancer, and to the best of our knowledge have never been investigated in AD, where protein aggregation is directly tied to pathology.

In addition, there are 50–500,000 insults to nuclear DNA in every cell as a result of normal metabolism every day [18] and as we age, the cell’s ability to handle the damage is diminished. Accumulation of DNA damage can cause destabilization of the genome, interference with DNA expression, cell senescence, and apoptosis [22]. In AD, excessive DNA damage [22, 76, 104], altered DNA repair [49, 94], chromosomal abnormalities [98], senescence [123], lethal cell-cycle re-entry [2, 79, 121] and altered neuronal death [23, 24, 32, 68] have all been reported.

In this study, we assessed the possibility of p53 aggregation in AD and AD-associated mouse models. We also assessed p53 for characteristics of other aggregation-prone proteins implicated in AD, such as seeding, cross-seeding, and toxicity. Lastly, we assessed if p53 aggregation may be detrimental to the p53-DNA damage pathway. Here, for the first time, we report evidence of p53 aggregation and contributions to pathology in human AD.

## Materials and methods

### Animals

This study was conducted in a facility approved by the American Association for the Accreditation of Laboratory Animal Care, and all experiments were performed in accordance with the National Institutes of Health *Guide for the Care and Use of Laboratory Animals* and approved by the Institutional Animal Care and Use Committee of the University of Texas Medical Branch. C57BL/6 J (The Jackson Laboratory #000664), Tg2576/Tau P301L (P301L) (Taconic #2469), Tau knockout (KO) (The Jackson Laboratory #007251), and hemizygous human tau htau mice (The Jackson laboratory stock #005491) [3] were bred at UTMB. Mice were housed at the UTMB animal care facility and maintained according to the U.S. Department of Agriculture standards (12-h light/dark cycle with food and water available ad libitum).

### Human tissue

Frozen Alzheimer’s disease and age-matched control frontal cortex brain tissue were obtained from the University of Kentucky Alzheimer’s Disease Center Tissue Bank (University of Kentucky Lexington, KY, USA). Brain tissue was collected with patient consent and protocols were approved by the Institutional Review Board of the University of Kentucky. All samples were examined by neuropathologists for diagnosis.

### Immunofluorescence (IF) for frozen mouse and human tissue

Frozen sections used for immunofluorescence were fixed in 100% chilled methanol, washed three times in 1X PBS, incubated in autofluorescence and lipofuscin eliminator TrueBlack (Biotium #23007) for 5 min, washed three times in 1X PBS, and blocked in 5% goat serum for 1 h. Sections were labeled with antibodies: total p53 (human tissue: rabbit anti-total p53 Abcam #32389 or mouse anti-total p53 Abcam #1101; Mouse tissue: mouse anti-total p53 Abcam #ab26), mouse anti-phosphorylated p53 (ser15) (Cell Signaling #9286), mouse anti-total tau (Tau13 Biolegend #835204), rabbit anti-I11 (oligomer) [55], rabbit anti-OC (fibrils; Millipore Sigma #ab2286), rabbit anti-T22 (tau oligomer; Millipore Sigma #ABN454) and incubated overnight at 4 °C. The following day, sections were washed in PBS three times for 10 min each and incubated with goat anti-mouse IgG Alexa-488 (1:500; Invitrogen) for 1 h. Sections were then washed in PBS three times for 10 min each and mounted with Prolong Gold containing DAPI. The sections were examined using a Keyence BZ-710 Microscope.

### Proximity ligation assay (PLA)

PLA in human brain tissue was performed using Duolink® PLA in Situ Red starter kit mouse/rabbit (Sigma Aldrich #DUO92101) per manufacturer’s protocol. Concentration of antibodies was established from IF protocol. Primary antibodies used for in–situ proximity assay include: rabbit anti-total p53 (Abcam #32389) and mouse anti-total tau (Tau13 Biolegend #835204). Amplified red signal was detected and imaged using Keyence BZ-710 Microscope.

### Purification of human recombinant p53

The plasmid pet15b/p53 (#24865) [5] containing the cDNA of human N-terminally His-tagged full-length p53 was transformed into *Escherichia coli* strain BL21(DE3). The resulting bacteria were grown at 37 °C to an OD_600_ = 0.4–0.6 before 3–4 h induction at 37 °C with 0.5 mM isopropyl β-D-thiogalactosidase (IPTG). After induction, cells were harvested by centrifugation and pellets stored at − 20 °C until use. The cell pellet was resuspended with 10 mL of column buffer (TritonX 100, 1 M Tris HCl (pH 8.0), 1 M NaCl, 1 M imidazole, and deionized water) containing protease inhibitor and then sonicated for 5 bursts for 30 s each on ice. Sonicated cells were then centrifuged at 10,000 RPM for 10 min by Ultracentrifuge. Purification was performed using chromatography columns: the resin was washed with deionized water 3x pellet volume and re-calibrated with 30 mL column buffer. Supernatant was incubated at RT with His-Pur Ni-NTA resin (Thermo Scientific #88221) for 1 h. Two elution fractions were collected using 50 mL of column buffer containing 250 mM (Elution 1) and 300 mM imidazole (Elution 2). Elutions were then dialyzed in 30 mM Tris HCl (pH 8.0) overnight to remove imidazole. Dialyzed elutions were then placed in 30 kD Amicon Ultra centrifugal filters (Millipore) to remove any degraded p53 protein and any residual imidazole. p53 elution concentration was determined by measuring A280 nm using Nanodrop and then confirmed by Western blot with a total p53 monoclonal antibody (Abcam #1101). The protein was either used immediately for the experiments or lyophilized using Labconco Benchtop Freeze Dryer and kept at − 80° for long term storage.

### Western blot

Mouse and human tissues were homogenized in buffer containing: 50 mM HEPES (pH 7.4), 150 mM NaCl, 12 mM β-glycerophosphate, 1 mM EGTA, 2 mM sodium orthovanadate, 1 mM NaF, 1 mM PMSF, 1% TritonX, 10 μg/ml of each of the following aprotinin, leupeptin, and pepstatin A. Brains were homogenized using a 1:3 dilution of tissue: buffer (w/v) using Qiagen TissueLyser LT and then centrifuged at 10,000 rpm for 10 min at 4 °C. Western blot was performed as previously described [74] with the exception that samples were heated at 95 °C for 10 min and/or heated with 2% β-mercaptoethanol. Fifty μg protein from brain homogenates was loaded. Primary antibodies used: total p53 human tissue (Abcam #1101), total p53 mouse tissue (Abcam #ab26), acetylated p53 (Abcam #75754), total histone H2AX (Abcam #124781), phosphorylated histone H2AX (cell signaling #2577), P53BP1 (Abcam #175188), p53R2 (Abcam #8816), MDM2 (Abcam #259265), and β-Actin Peroxidase (Sigma A3854).

### Size exclusion chromatography (SEC) on fast protein liquid chromatography (FPLC)

Recombinant human p53 samples were separated by SEC using Amersham Biosciences AKTA explorer FPLC system fitted with Superdex 200 10/300 Increase GL column (tricorn). L × I.D. 10 × 300 mm, 13 μm particle size from GE Healthcare as previously described [74]. Molecular grade water was used as the mobile phase, flow rate 0.5 mL/min. Gel filtration standard (Bio-Rad 51–1901) was used for calibrations. Excitation and emission wavelengths used for absorbance detection were 280 nm and 350 nm, respectively. Desired p53 monomer and oligomer peaks were collected and tested by western blot and AFM for further confirmation.

### Atomic force microscopy (AFM)

The morphology of p53 monomers and oligomers and fibrils were assessed by AFM as previously described [37, 64, 73]. Briefly, samples were prepared by adding 10 μL of p53 monomer or oligomers on freshly cleaved mica and allowed to adsorb to the surface. Mica was then washed three times with distilled water to remove unbound protein and impurities followed by air-drying. Samples were then imaged with a Multimode 8 AFM machine (Veeco, CA) using a noncontact tapping method (ScanAsyst-Air).

### Tau oligomer (tauO) and p53 oligomer (p53O) production, labeling, and cell treatments

tau oligomer (tauO) and p53O were produced and characterized following established and published protocols [36] with an exception that recombinant p53 was heated as previously described [34]. tauO and p53 oligomer (p53O) labeling was conducted as follows: 1 mg of Alexa Fluor™ 568 NHS Ester (Invitrogen, #A20003) was dissolved in 0.1 M sodium bicarbonate to make the final concentration 1 mg/ml. The Alexa-Fluor dye was then incubated with tauO and p53O in a 1:2 (w/w) ratio. The mixture was rotated overnight at 4 °C on an orbital shaker. The following day, the solution was centrifuged (30 min, 15,000 *g*) using 10 kDa Amicon Ultra-0.5 Centrifugal Filter Units to remove unbound dye. The oligomers were then washed with 1× PBS until the flow-through solution was clear. The filter compartment was then flipped and centrifuged to collect the concentrate. The oligomers were reconstituted to their original volume. Alexa-Fluor labeled tauO and p53O were re-suspended in complete DMEM to obtain 0.5 or 1 μM final concentration solutions. The cells were treated with tauO or p53O for 1 or 4 h timepoints at a controlled temperature of 37 °C and 5% CO_2_. Afterwards, the medium was removed, and the cells collected for immunofluorescence assays.

### Primary cortical neuron culture

Primary cortical neuronal cultures were prepared and maintained as described previously [7, 74, 84, 92]. Briefly, cortical neurons were isolated from embryonic day 16–18 C57BL/6 mice (The Jackson Laboratory #000664) using Accutase® solution (A6964-100Ml Sigma-Aldrich). Dissociated neurons were plated at a density of 2 × 10^5^ cells/ml in a 24-well plate containing high glucose DMEM (Corning) supplemented with 2% B27 (A3582801, Gibco), 10,000 units/ml penicillin, 10,000 μg/ml streptomycin, and 25 μg/ml amphotericin B (15,290,018, Gibco). After 2 h, plating medium was removed from cells and replenished with Neurobasal™ medium (12,348,017, Gibco) plus 2% B27, 0.5 mM L-glutamine (SH30034.01, HyClone), 10,000 units/ml penicillin, 10,000 μg/ml streptomycin, and 25 μg/ml amphotericin B supplement. 50% of medium changes were performed every 3–5 days. Cells on day in vitro (DIV) 10 were used for all experiments.

### Immunofluorescence of fixed C57Bl/6 primary neurons and confocal microscopy

Primary cortical neuronal cultures derived from C57Bl/6 and Tau KO mice (Tau KO primary neurons only used for LDH) were prepared and maintained as previously described [74, 84, 92]. The primary antibodies used in this study for immunocytochemistry are as follows: rabbit anti-I11 [55], mouse anti-β-III-Tubulin (Abcam #78078), rabbit anti-total p53 (Abcam #246550), rabbit anti-total tau (Abcam #64193), mouse anti-phosphorylated p53 (Ser15) (Cell Signaling #9284), and rabbit anti-phosphorylated histone H2AX (Ser139) (Cell Signaling #2577). After three washes with PBS, cells were probed with mouse and rabbit-specific fluorescent-labeled secondary antibodies (1:200, Alexa Fluor 568, Life Technologies). The single-frame images and Z-stacks for orthogonal view were collected using a Keyence Confocal Microscope. To build the Z-stack, 17 stacks/0.3–0.4-μm optimal thickness were captured. Each treatment condition was randomly imaged in five different regions of interest and performed in duplicate. Imaging processed with ImageJ Software (NIH).

### LDH

Primary neurons derived from C57BL/6 and Tau KO mice were cultured and treated for measuring cytotoxicity using LDH release assay (Cytotoxicity Detection Kit PLUS-LDH, #04744926001, Roche) following the manufacturers’ instructions as previously described [74, 102]. Briefly, primary neurons were maintained in Neuroblast medium supplement with B-27. Primary neurons (3 × 10^5^ cells /well) derived from C57BL/6 (*n* = 2) and tau KO (*n* = 1) were treated for 24 h with 0.5 μM or 1.0 μM p53 monomer, p53 oligomer, p53 fibrils, and p53 mixtures (each treatment performed in triplicate) and assessed by LDH release. OD was measured at 490 nm with POLARstar OMEGA plate reader (BMG Labtechnologies).

### Statistical analysis

The number of replica wells and experiments were indicated in the figure legends for each assay when appropriate. Densitometry from western blot was performed using ImageJ software. Fluorescence intensity and colocalization measurements, including Pearson’s Correlation Coefficients and intensity scatterplots, from immunofluorescent staining were also performed using ImageJ. Values were placed into GraphPad Prism 8.0 (GraphPad Software, Inc., SanDiego, CA, USA) for statistical analysis. Unpaired and Two-tailed (α = 0.05) Student’s T-Test were performed where appropriate. Data are presented as the mean ± SEM. One-way ANOVA with Bonferroni Post Hoc test was also used where appropriate. Grubbs test was used to eliminate any outliers with an α = 0.05. Results considered significant as follows *p < 0.05, **p < 0.01, *** *p* < 0.001, **** *p* < 0.0001.

## Results

### p53 oligomers and fibrils detected in the frontal cortex of human AD brain, but not age-matched controls

**Fig. 1:**
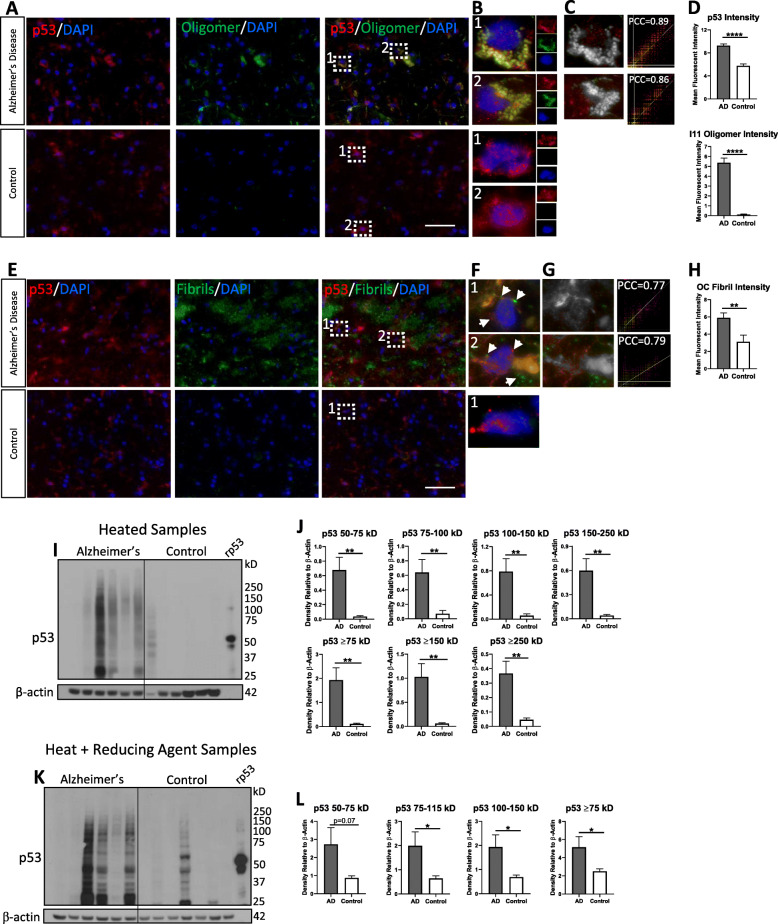
p53 oligomers and fibrils detected in frontal cortex of human AD brain, not age-matched controls. **a** Representative confocal images of human AD and age-matched control brain immunofluorescently labeled with anti-p53 (red) and anti-I11 (green; oligomer-specific). **b** *Left* Magnified ROI from merged panels in A. Colocalization between p53 and I11 is shown in the AD tissue, but not in control tissue. **c** *Middle:* colocalized pixel maps show peri-nuclear overlap of signal with other oligomers and p53 in vicinity. *Right:* Intensity scatterplot with PCC of 0.86–0.89 indicate a high degree of colocalization suggesting that p53 oligomers occur in AD brain. No colocalized pixel map or PCC calculated for control as there is no detected colocalization. **d** Fluorescent intensity calculated from AD and control brain for p53 (*n* = 4 Ctrl; *n* = 7 AD in technical triplicate) and I11 (*n* = 4 Ctrl; *n* = 3 AD in technical triplicate). **e** Representative confocal images of human AD and age-matched control brain immunofluorescently probed with anti-p53 (red) and anti-OC (green; fibril-specific). **f** *Left* Magnified ROI from merged panels in E. Colocalization between p53 and OC is depicted in the AD tissue, but not in control tissue. **g** *Middle:* colocalized pixel maps show peri-nuclear overlap of signal with other fibrils and p53 in vicinity. *Right:* Intensity scatterplot with strong PCC, suggesting that p53 fibrils occur in AD brain. No colocalized pixel map or PCC calculated for control as there is no detected colocalization. **h** Fluorescent intensity calculated from AD and control brain tissue for OC (*n* = 4 Ctrl and *n* = 6 AD in technical triplicate). Keyence Microscope 60X Magnification. Scale bar = 50 μm. **i** AD and control brain samples heated and then probed by Western blot for p53 (rp53 = recombinant p53 control). **j** Densitometry analysis of different p53 bands (*n* = 6 in technical duplicate). **k** AD and control brain samples heated with reducing agent and then probed by Western blot for p53. **l** Densitometry analysis of different p53 bands (*n* = 7 Ctrl; *n* = 6 AD)

Human AD frontal cortex (Braak stage 6) and age-matched control patients (Braak stage 0–1) (Supplemental Table 1) were analyzed by immunofluorescence for total p53 and oligomers using I11, a polyclonal antibody that detects the conformation of oligomers formed by a variety of aggregation-prone proteins [55] and evaluated by confocal microscopy. Colocalization between p53 and I11 oligomers are shown in AD brain, but not in control brain (Fig. 1a). Magnified regions of interest with colocalized pixel maps demonstrate the peri-nuclear overlap of signal in AD tissue with other oligomers and p53 in the vicinity (Fig. 1b-c). Intensity scatterplots with strong Pearson’s Correlation Coefficient (PCC) values also indicate a high degree of colocalization between fluorophores, suggesting p53 forms oligomeric conformations in AD brain.

To investigate if p53 fibrils could be detected in AD, we performed immunofluorescence with OC, a conformational, polyclonal antibody that recognizes protein fibrils. Colocalization between p53 and OC fibrils are shown in AD tissue, but not in control tissue (Fig. 1e). Magnified regions of interest (Fig. 1f) with colocalized pixel maps (Fig. 1g) demonstrate peri-nuclear overlap of signal with other fibrils and non-colocalizing p53 in the vicinity. Intensity scatterplots with strong PCC values also indicates a high degree of colocalization between fluorophores, suggesting p53 fibrils can also be detected in AD brain. Fluorescence intensity for total p53, total I11, and total OC fibrils all demonstrate a significant increase in AD brain compared to controls (Fig. 1d, h). An increase in the amount of total p53 in AD brain (Fig. 1d) supports previous observations [16, 28, 57, 88]. Western blot of human cortical brain tissue demonstrates high molecular weight p53 above 53 kD that is resistant to heat (Fig. 1i), denaturing and reducing conditions (Fig. 1k) in AD brain, demonstrating that these are likely not complexes of p53, but possibly oligomers. Densitometry demonstrates significantly more p53 signal in AD brain compared to control at 50–75 kD, 75–100 kD, 100–150 kD, 150–250 kD, ≥75 kD, ≥150 kD, and ≥ 250 kD when samples are heated (Fig. 1j). Of note, although a consistent result was found in most control brains, one sample of seven, did present as an outlier and may be indicative of the wide variability seen in aged humans (Fig. 1k). Densitometry demonstrates significantly more p53 signal in AD brain compared to control at 75–115 kD, 100–150 kD, and ≥ 75 kD when samples are heated and reduced (Fig. 1l). It is important to note a limitation of separation by gel electrophoresis, as it may alter the size of aggregates (e.g. promoting the release of oligomers from fibrils) and thus may not accurately reflect the true p53 aggregate size. Bands below the p53 monomer at 53 kD were also detected. Although we are not certain, these bands may represent p53 isoforms [12] and/or p53 cleavage products [6, 80, 101]. Together, this data suggests that p53 oligomers and fibrils are present in AD brains.

### Phosphorylated p53 forms oligomers and is mislocalized in the frontal cortex of human AD brain, but not age-matched controls

**Fig. 2:**
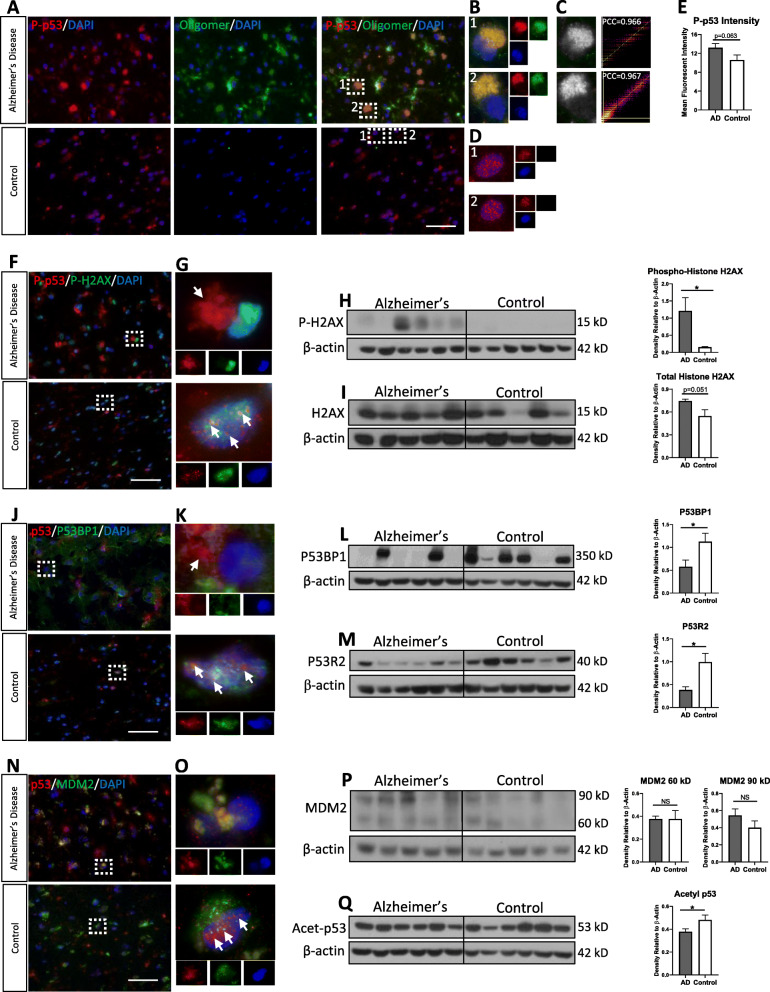
Phosphorylated p53 forms oligomers, mislocalizes, and DNA damage response is impaired in human AD brain. **a** Representative confocal images of human AD and age-matched control brain probed with anti-P-p53 antibody (red) and anti-I11 antibody (green; oligomer-specific). **b** Magnified ROI from AD merged panels in A. Colocalization between P-p53 and I11 is shown in the AD tissue with other oligomers in vicinity. **c** Colocalized pixel map and intensity scatterplot with PCC of 0.96 indicates a high degree of colocalization, suggesting P-p53 oligomers form in AD brain. **d** Magnified ROI from control merged panel in A. No colocalization detected in control brain. **e** Fluorescent intensity of P-p53 in AD and control brain shows no significant difference (*n* = 3 in technical triplicate). **f** Representative confocal images of human AD and control brain probed with anti-P-p53 (red) and anti-P-H2AX (green) in merge panel. **g** Magnified ROI from AD merged panels in F. Control brain shows P-p53 in nuclei with P-H2AX positive signal. **h** Western blot probed for total histone H2AX and (**i**) P-H2AX (*n* = 6 in technical duplicate). **j** Representative confocal images of human AD and control brain probed with anti-p53 (red) and anti-p53BP1 (green) in merge panel. **k** Magnified ROI from AD merged panels in J. **l** Western blot probed for P53BP1(*n* = 6 in technical duplicate), and P53R2 (*n* = 6 in technical duplicate) (**m**) show significant decrease in AD brain. **n** Representative confocal images of human AD and age-matched control brain immunofluorescently probed with anti-p53 (red) and anti-MDM2 (green) in merge panel. **o** Magnified ROI from AD and control merged panels in N. **p** Western blot of AD and control brain probed for MDM2 show no significance difference at the full-length (90kD) and caspase-3 fragment (60 kD) (*n* = 5). **q** Western blot probed for acetylated-p53 shows a significant decrease in AD brain (*n* = 6 in technical duplicate). Immunoblot probed with β-actin shown in L was reused in Q. Keyence Microscope 60X magnification. Scale bar = 50 μm

Next, we wanted to assess if oligomerization affected p53 phosphorylation. p53 is normally phosphorylated at Ser15 in response to DNA damage, causing it to dissociate from its inhibitor MDM2, and translocate to the nucleus. Human frontal cortex from AD and age-matched control patients were stained with immunofluorescent markers for phosphorylated p53 (P-p53) at Ser15 and oligomers with I11 (Fig. 2a). Colocalization (depicted in yellow) between P-p53 and I11 is found in the cortical AD brain but not cortical control brain. Regions of interest show peri-nuclear colocalization with non-colocalizing P-p53 and unidentified oligomers present in the vicinity (Fig. 2b). A colocalized pixel map and strong PCC support this colocalization (Fig. 2c), suggesting P-p53 also forms oligomers in AD. Conversely, in control tissue, P-p53 forms distinct puncta in the nucleus (Fig. 2d). There is no significant difference in P-p53 fluorescence intensity between AD and control brain tissue (Fig. 2e), however, subcellular localization is different. Together, these data suggest that P-p53 forms oligomers and are mislocalized, outside the nucleus, in AD brain.

### p53-regulated mediators of DNA damage are reduced in AD despite evidence of DNA damage

To investigate DNA damage, we probed human control and AD cortical brain with P-p53 (Ser15) and phosphorylated (Ser139) histone H2AX (P-H2AX) also known as γH2AX, a marker for DNA double-strand breaks (DSBs) [75, 97] (Fig. 2f). DNA damage response is intact in control brain, with P-p53 and P-H2AX both forming distinct puncta in nuclei (Fig. 2g, bottom panel). However, P-H2AX-positive neurons in AD do not show P-p53 signal inside the nucleus (Fig. 2g, top panel). In AD, P-p53 does not form puncta and is located outside the nucleus, possibly due to its aggregation. Western blot of AD and control brain demonstrate a significant increase in phosphorylated levels of Histone H2A.X and nearing significance (*p* = 0.051) in total levels of Histone H2A.X (Fig. 2h-i). This indicates a significant increase in DNA DSB damage in AD brain over controls. A 2-fold increase in DSBs [87] and excessive DNA damage outside normal aging has been reported in AD [22, 76, 104]. Densitometry from Western blot of human AD and control brain also demonstrates a significant decrease in acetylated (K382) p53, another p53 post-translational modification (PTM) following DNA damage that enhances p53 transcriptional activity (Fig. 2q). Furthermore, similar results were demonstrated by confocal imaging with P53 binding protein 1 (P53BP1) (Fig. 2j), which is a direct responder to DNA double-strand breaks and also binds p53 at its DNA-binding domain for more efficient p53-mediated transcriptional activity [1, 48]. In control brain, P53BP1 is clearly inside the nucleus with p53, but not in AD brain (Fig. 2k). Western blot analysis also demonstrates a significant decrease of P53BP1 in AD despite evidence of increased DNA damage (Fig. 2l).

Downstream DNA damage responders transcriptionally regulated by p53, such as p53-inducible ribonucleotide reductase small subunit 2 (P53R2), also shows significant reduction by Western blot in AD brain compared to control (Fig. 2m) despite strong indication of DNA damage. P53R2 directly responds to DNA DSBs, translocating to the nucleus where it provides deoxyribonucleotides locally for DNA repair and is also responsible for mitochondrial DNA synthesis. It is also important to note that p53R2 is the primary small subunit of the ribonucleotide reductase in post-mitotic cells [17]. Therefore, both nuclear and mitochondrial DNA integrity may be impaired due to loss of p53 function in AD. Overall, this suggests a breakdown in the p53-mediated DNA damage response pathway, which may be linked to p53 aggregation and subsequent cytoplasmic sequestration, contributing to nuclear p53 loss of function.

The major inhibitor and E3 ubiquitin ligase of p53 is mouse double minute 2 homolog (MDM2) [61, 83, 90]. Western blot analysis of MDM2 with human AD and control brain demonstrates no significant difference in total MDM2 levels at the full-length (90kD) or caspase-3 apoptotic fragment (60 kD) (Fig. 2p). Confocal imaging of total p53 and MDM2 shows colocalization of p53 and MDM2 outside the nucleus in AD (Fig. 2n and o**-**top panel). This may suggest MDM2 is inhibiting p53 or ubiquitinating p53 for degradation by the 26 s proteasome. However, it remains to be seen if MDM2 binds aggregated p53. In contrast, human control brain demonstrates no colocalization, with a majority of p53 appearing to overlap with the nucleus and a majority of the MDM2 appearing outside the nucleus (Fig. 2n and o**-**bottom panel). Overall, this would suggest that p53 is active in control brain while AD brain appears to lack a robust p53 response.

### p53 interacts with tau in human frontal cortex; tau oligomers interact with p53 in human AD brain, but not age-matched controls

**Fig. 3:**
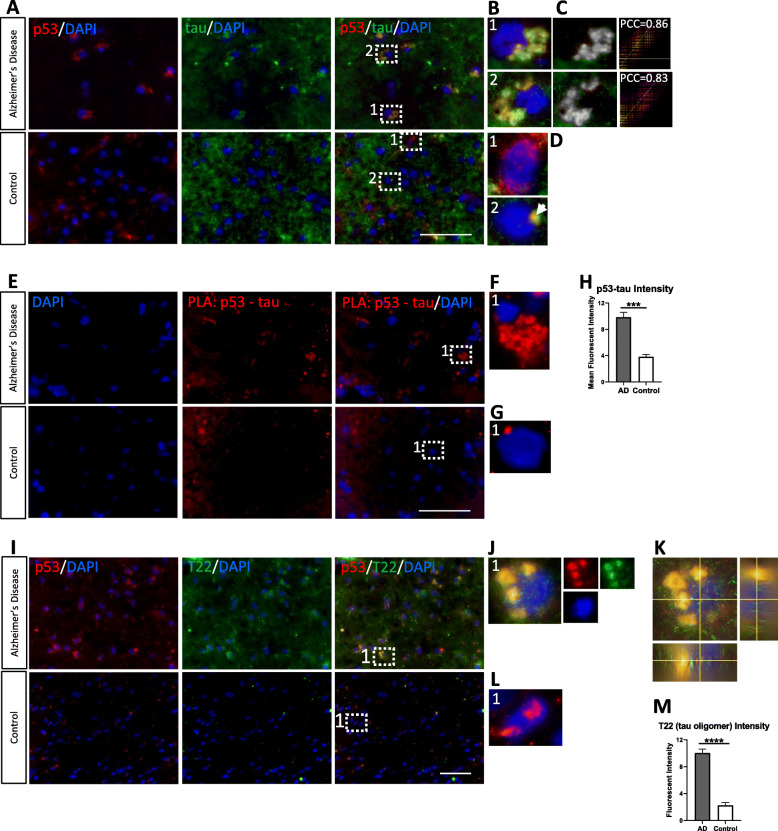
p53 interacts with tau in human brain; tau oligomers interact with p53 in AD brain. **a** Representative confocal images of human AD and age-matched control brain immunofluorescently probed with anti-p53 (red) and anti-tau (green). **b** Magnified ROI from AD merged panels in A. Large peri-nuclear colocalization between p53 and tau is shown in AD brain with other tau in the vicinity. **c** Colocalized pixel map of signal and intensity scatterplot with strong PCC (0.83–86). **d** Magnified ROI from control merged panels in A. (1) Diffuse p53 signal with very little colocalization shown. (2) Small single peri-nuclear region of colocalization detected in control brain. **e** Representative confocal images of human AD and control brain using PLA with anti-p53 and anti-tau (Tau13 Biolegend). **f** Magnified ROI from AD merged panel in E shows large peri-nuclear interaction. **g** Magnified ROI from control merged panel in E shows single small region of colocalization similar to magnified image in D. **h** Fluorescent intensity of p53-tau from PLA shows significant increase in p53-tau interaction in AD brain (*n* = 2; in technical triplicate). **i** Representative immunofluorescence confocal images of human AD and control brain probed with anti-p53 (red) and anti-T22 (tauO specific; green). **j** Magnified ROI from AD merged panels in I. Large peri-nuclear colocalization between p53 and tauO are shown in AD brain with other tau in the vicinity. **k** Orthogonal view of magnified ROI in J shows a large amount of p53 is localized outside the nucleus where it heavily colocalizes with T22. TauO is also observed inside the nucleus by orthogonal view. **l** Magnified ROI from control merged panel in I. p53 is observed in the nucleus and no tauO are observed in control brain. **m** Fluorescent intensity of T22 shows significantly more tau oligomers in AD brain (*n* = 3 in technical triplicate). Keyence Microscope 60X-100X magnification. Scale bar = 50 μm

Next, we wanted to explore why p53 is aggregating, mislocalized, and not responding to lethal DNA damage. It is possible that p53 nucleo-cytoplasmic transport could be disrupted and since microtubules assist with transport and tau, a microtubule associated protein, is well known to cause pathology in AD, we wanted to understand if there was any influence from a disruption in tau. Human frontal cortex from AD and age-matched controls were probed with immunofluorescent markers for total p53 and total tau and evaluated by confocal imaging (Fig. 3a). Magnified regions of interest from AD brain show large peri-nuclear colocalization with no detectable p53 overlapping with the nucleus (Fig. 3b). Colocalized pixel maps with strong PCC (Fig. 3c) firmly suggest an interaction between p53 and tau. Conversely, control brain shows some neurons with diffuse p53 signal (Fig. 3D1) and other neurons with a single small region of colocalization between p53 and tau (Fig. 3D2). This would suggest that p53 may normally interact with WT tau in the human cortex, but the interaction is more common and widespread in AD with pathological tau. Proximity Ligation Assay (PLA) using the same total p53 and total tau antibodies and human tissue demonstrates a similar pattern of colocalization seen by immunofluorescence (Fig. 3e-g). Of note, this PLA only causes fluorescence when the fluorophores are within 40 nm of each other, a strong indication of direct physical interaction rather than the two proteins merely being in the same subcellular region. Fluorescence intensity from the PLA demonstrates a significant increase in p53-tau intensity in AD brain compared to control brain (Fig. 3h). Together, this suggests that there is an interaction between p53 and tau in human cortex with a larger degree found in AD than control brain.

As we were able to show an interaction between p53 and tau, we also wanted to determine if tau oligomers (tauO) were part of the total tau species previously observed, as tauO have been shown to cause toxicity and cross-seed other proteins in AD [19, 20, 31, 46, 53, 64, 67, 86, 113]. Immunofluorescence in human AD and control brain for total p53 and tauO with T22 (polyclonal antibody that specifically recognizes tau oligomers) show peri-nuclear colocalization in AD but not control brain (Fig. 3i). Magnified regions of interest (ROI) show large areas of colocalization and highlight other tauO in the vicinity (Fig. 3j). Conversely, magnified ROI in the control tissue show p53 signal overlapping with the nucleus and no tauO (Fig. 3l). Orthogonal view of the ROI in AD brain demonstrates that a majority of the p53 is outside the nucleus, heavily colocalizing with tauO. Orthogonal view of AD brain also shows detectable tauO in the nucleus, which may be causing unknown pathology (Fig. 3k). Fluorescent intensity of confocal images demonstrates a significant increase in tauO in AD brain (Fig. 3m). Overall, we may conclude that there is an interaction between p53 and tauO in AD brain that is not found in controls and this interaction may play a role in p53 sequestration or aggregation outside the nucleus.

### p53 oligomers detected in brain tissue of aged tau mouse models but not in aged C57Bl/6 or tau KO mice

**Fig. 4:**
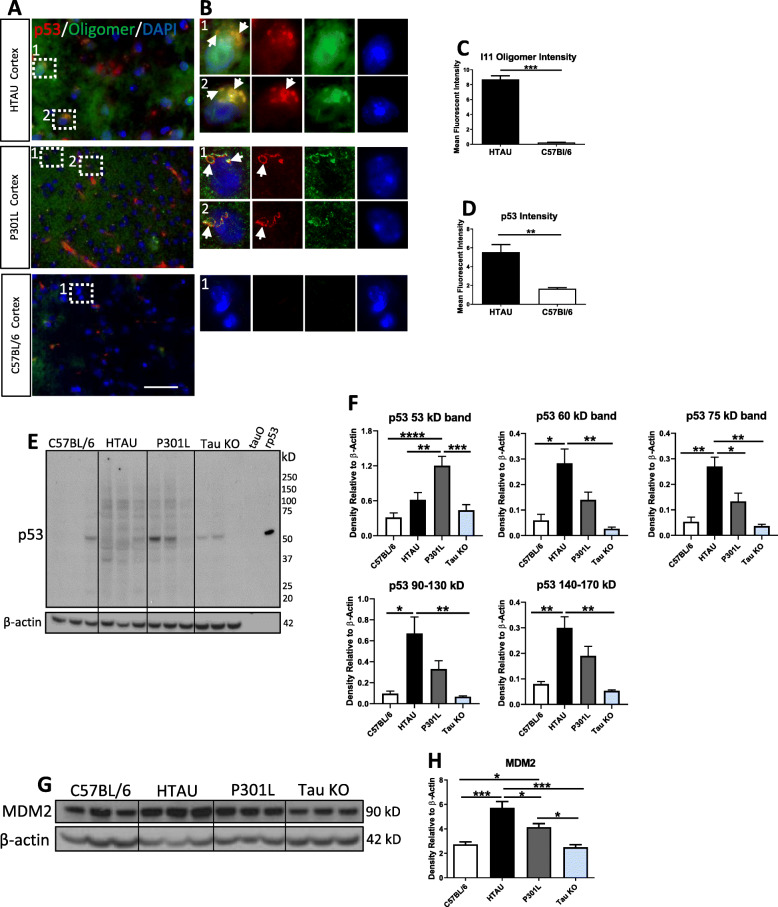
p53 oligomers detected in aged tau mouse models, but not in aged control mice. **a** Representative immunofluorescent confocal images of cortex of 14-month-old htau, 5-month-old P301L, and 9-month-old C57BL/6 mice. Brain tissue probed with anti-p53 (red) and anti-I11 (green; oligomer-specific) in merge panel. **b** Magnified ROI from merged panel in A. (htau ROI 1 and 2) Peri-nuclear colocalization between p53 and I11 is shown with other oligomers in the vicinity. (P301L ROI 1 and 2) Peri-nuclear, circular colocalization between p53 and I11 is shown with other oligomers in the vicinity. (C57BL/6 ROI 1) No colocalization between p53 and I11 is detected in C57BL/6 mice. Keyence Microscope 60X magnification. Scale bar = 50 μm. **c** Graph depicting immunofluorescent intensity for I11 oligomer (*n* = 2 in technical triplicate) and (**d**) p53 intensities from htau and C57BL/6 mice (*n* = 2 in technical triplicate). Keyence Microscope 60X magnification. Scale bar = 50 μm. **e** Western blot of aged C57BL/6, htau, P301L, and Tau KO mice probed with anti-p53. **f** Densitometry of p53 bands shows a significant increase in p53 in htau mice at multiple bands compared to C57BL/6 and Tau KO mice (*n* = 3). **g** Western blot of same mice probed with anti-MDM2 (90 kD). **h** Densitometry of MDM2 from G shows significant increase in MDM2 in htau compared to all other mouse models. P301L also show significantly more MDM2 compared to control and Tau KO mice. (*n* = 3 in technical duplicate)

After observing an influence of tau on p53 in the human brain, we wanted to assess whether tau transgenic mouse models may also show similar p53 oligomer formation. The htau mouse model overexpresses all 6 isoforms of human tau and develops neurofibrillary tangles at 15 months of age with major memory deficits observed by 12 months of age [3]. Tg2576/Tau (P301L) transgenic mice express human APP with the Swedish mutation and human tau (4R/0 N inserts) with the P301L mutation. These mice develop plaques and NFT near 6 months of age [72]. C57Bl/6 and Tau KO mice were used as controls. Cortex from 14-month-old htau mice, 5-month-old P301L mice, and 9-month-old C57Bl/6 mice were probed with antibodies against total p53 and I11 (Fig. 4a). Peri-nuclear colocalization between p53 and I11 is shown in htau and P301L mice, but not C57Bl/6 mice and can be more clearly seen in magnified ROI (Fig. 4b). Interestingly, colocalization in a circular pattern was noted in both htau and P301L mice and may represent unknown pathology that will require further study (Fig. 4b, white arrows). Moreover, confocal intensity demonstrates a significant increase in both oligomers and p53 in htau mouse brain compared to C57Bl/6 mice (Fig. 4c-d).

Furthermore, Western blot analysis of all four mouse models (Fig. 4e) containing aged mice (C57Bl/6 age:17 months; htau age 13, 14, 19 months; P301L age 16 months; Tau KO age 16 months) demonstrated a significant increase in p53 monomer in P301L mice compared to all other mouse groups (Fig. 4f). Aged htau mice show significant increase in p53 bands detected at 60 kD, 75 kD, 90–130 kD, and 140–170 kD compared to aged C57Bl/6 mice and aged Tau KO mice (Fig. 4f), suggesting that tau overexpression causes high molecular weight p53 to form. These same bands are detected in P301L mice, but not C57Bl/6 or Tau KO mice, although they are not significant by densitometry. MDM2 levels in mouse brain were also assessed by Western blot (Fig. 4g) and demonstrate significantly more MDM2 in the htau and P301L mice as compared to both C57Bl/6 and Tau KO control mice (Fig. 4h). As MDM2 is a negative regulator of p53, it is unsurprising that mouse brain with more p53 also contains more MDM2 to help downregulate p53 activity. Together, this indicates that p53 oligomers can be found in the cortex of aged htau and P301L mice, but not in control mice and suggests that tau pathology may influence p53 aggregation.

### p53 oligomers colocalize with WT p53 and may represent seeding. Tau oligomers colocalize with WT p53 and phosphorylated p53 and may represent cross-seeding

Due to the evidence of p53 oligomers and interactions between p53 and tauO, we wanted to understand how exogenous treatment of p53O or tauO may affect endogenous p53 and tau in neurons, specifically looking for evidence of seeding or cross-seeding. To do this, we produced purified human recombinant full-length p53 and created oligomers as previously described [54, 66] (Supplemental Figure 1). C57Bl/6 primary neurons were treated with exogenous, recombinant, Alexa-fluor-labeled p53 oligomers (AFL-p53O) at 1 μM concentration for 1 h and 4 h. To determine uptake and internalization of p53O, we stained primary neurons (both treated with AFL-p53O and untreated) with antibodies against total p53 and the neuronal membrane marker, β-III-Tubulin (Supplemental Figure 2A-B). AFL-p53O are observed within the confines of the β-III-tubulin signal, suggesting that p53O are internalized by C57Bl/6 primary neurons within 1 h and are maintained inside the nucleus up to 4 h (Supplemental Figure 2B). Similar results with 1 μM p53 aggregates have been previously reported in cancer cell lines [34]. Alexa-fluor labeled tauO show the same internalization at two different concentrations (0.5 μM and 1 μM) and are taken up within 1 h and appear to remain inside the neuron up to 4 h (Supplemental Figure 2D).

**Fig. 5:**
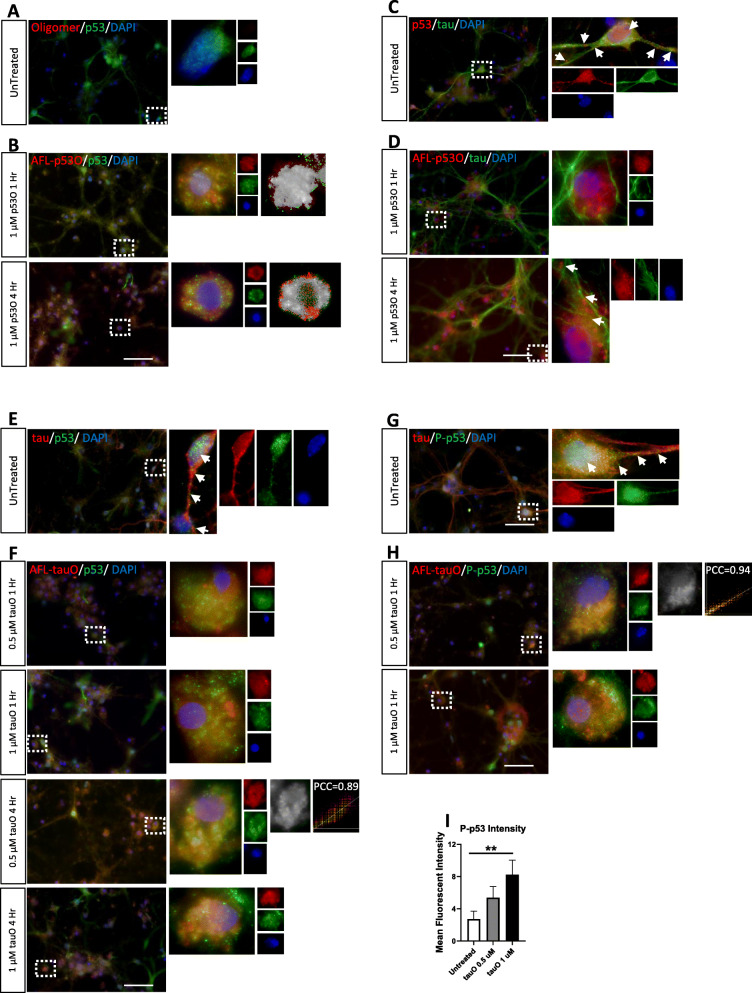
p53 oligomers colocalize with p53, but not tau. Tau oligomers colocalize with p53 and P-p53. **a** Representative confocal image of untreated C57BL/6 primary neurons probed with anti-p53 (green), and I11 (oligomer specific). Magnified ROI shows p53 signal in the nucleus and no oligomers. **b** Representative immunofluorescent confocal images of C57BL/6 primary neurons treated with AFL-p53O were probed with anti-p53 (green). Peri-nuclear colocalization between AFL-p53O and endogenous WT p53 is observed at 1 h and 4 h. Magnified ROI with colocalized pixel maps show colocalization between AFL-p53O and endogenous p53. **c** Representative confocal image of untreated C57BL/6 primary neurons probed with anti-p53 (red) and anti-tau (green). Magnified ROI shows p53 signal in the nucleus and on the microtubules. **d** Confocal Imaging of C57BL/6 primary neurons treated with AFL-p53O were immunofluorescent probed with anti-tau (red) and show no colocalization between tau and AFL-p53O. At 4 h, magnified ROI show peri-nuclear p53 signal that also appears to travel on the microtubules. **e** Representative confocal image of untreated C57BL/6 primary neurons probed with anti-p53 (green) and anti-tau (red). Magnified ROI shows p53 signal in the nucleus and on the microtubules. **f** Confocal Imaging of C57BL/6 primary neurons treated with AFL-tauO were immunofluorescently probed with anti-p53 (green) and show colocalization between AFL-tauO and endogenous p53. Magnified ROI show peri-nuclear colocalization at both concentrations and timepoints. **g** Representative confocal image of untreated C57BL/6 primary neurons probed with anti-P-p53 (green), and anti-tau (red). Magnified ROI shows P-p53 signal in the nucleus and on the microtubules. **h** Confocal Imaging of C57BL/6 primary neurons treated with AFL-tauO were immunofluorescently probed with anti-P-p53 (green) and show colocalization between AFL-tauO and endogenous P-p53. Magnified ROI show peri-nuclear colocalization at both concentrations at 1 h. **i** Fluorescent intensity of P-p53 shows a significant increase at 1 μM tauO concentration as compared to untreated. Keyence microscope, 60X magnification, scale bar = 50 μm

After confirming that p53O could indeed be taken up by primary neurons, we investigated if exogenous human p53O would interact with endogenous mouse p53, potentially seeding the endogenous p53. Untreated C57Bl/6 primary neurons demonstrate no evidence of endogenous oligomers and endogenous p53 localizes to the nucleus (Fig. 5a). In contrast, C57Bl/6 primary neurons treated with AFL-p53O were stained with an antibody against total p53 (specifically recognizes mouse, not human p53) and confocal imaging demonstrates colocalization between AFL-p53O and endogenous p53 up to 4 h in a peri-nuclear localization (Fig. 5b). This suggests that exogenous p53O localize and may interact with endogenous p53 (Fig. 5b), potentially allowing p53O seeding. Next, we stained p53O treated primary neurons with an antibody against total tau to determine if p53O may interact with endogenous tau. No colocalization was found, suggesting that p53O do not interact with WT tau for up to 4 h of treatment at a concentration of 1 μm of p53O (Fig. 5d). P53O may have different effects at higher, toxic concentrations. LDH assay shows p53O up to 1 μM do not cause toxicity through disruption of the cell membrane in primary neurons derived from C57BL/6 or Tau KO mice (Supplemental Figure 3), which has been reported by others [96]. Interestingly, p53 is observed far from the nucleus, along tau-positive microtubules in untreated cells as well as p53O after 4 h treatment (white arrows; Fig. 5c, e, d). This may suggest that WT p53 can be found on neuronal axons and that p53O may spread along the microtubules.

Next, we considered the reciprocal treatment, to determine if tauO could affect endogenous p53 in WT primary neurons. Therefore, we Alexa-fluor labeled tauO (AFL-tauO) and then treated C57Bl/6 primary neurons with AFL-tauO at 1 μM and 0.5 μM for 1 h and 4 h treatments. The 4 h treatment was chosen as previous publications have demonstrated tauO toxicity at 1 μM at 6 h [74] and we wanted to consider interactions before severe neuronal death occurred. Immunofluorescent staining of primary neurons with total p53 and Alexa-fluor labeled tauO demonstrate peri-nuclear colocalization between AFL-tauO and endogenous p53 at both 1 h and 4 h timepoints (Fig. 5f) that is not seen in untreated primary neurons (Fig. 5e). A colocalized pixel map and strong PCC (PCC = 0.89) support this colocalization, suggesting that tauO can interact with endogenous p53. Given past evidence of the ability of tauO to cross-seed [19, 20, 31, 46, 53, 100, 119], p53 could be another potential cross-seeding partner with serious ramifications for AD pathology.

Lastly, we wanted to understand how tauO treatment may affect the p53-DNA damage pathway, specifically if tauO can directly cause DNA damage, spurring p53 to become phosphorylated at Ser15 in response. We probed untreated and 1 h tauO treated (0.5 μM and 1 μM) C57BL/6 primary neurons with P-p53 (Fig. 5g-h) and P-H2AX (Supplemental Figure 2E-H). Remarkably, tauO was found to colocalize with P-p53 in the peri-nuclear region of primary neurons after just 1 h at two different concentrations (Fig. 5h). A colocalized pixel map and strong PCC (PCC = 0.94) support this colocalization, suggesting that tauO can also interact with endogenous phosphorylated p53. Fluorescent intensity of the confocal images shows a significant increase in P-p53 levels after 1 h of a 1 μM tauO treatment compared to untreated (Fig. 5i). Moreover, confocal imaging with P-H2AX, a marker for DNA double- strand breaks, shows puncta in the nucleus of untreated (Supplemental Figure 2F) and treated primary neurons, however, to a much stronger degree in the neurons that received tauO treatment (Supplemental Figure 2H). Indeed, P-H2AX levels were significantly increased following tauO treatment at both 0.5 μM and 1 μM compared to primary neurons that were untreated by confocal fluorescent intensity (Supplemental Figure 2I). Overall, this suggests that tauO may indirectly increase DNA damage, leading to P-p53 up-regulation that may be disrupted by interactions with tauO.

## Discussion

### p53 forms oligomers and fibrils in Alzheimer’s disease

p53 aggregates, derived from fragments [4, 47, 96] and full-length p53 [66], have been reported in human disease since 1996 [82]. We hypothesized that p53 may also aggregate in AD, a disease characterized by accumulation of protein aggregates. Our data presented here demonstrates: 1) p53 forms oligomers and fibrils in AD, 2) DNA damage repair pathways regulated by p53 are impaired in AD, 3) WT p53 and tauO interact in AD and in vitro using exogenous oligomer treatments providing evidence for cross-seeding, 4) p53O can be observed in tau transgenic mouse models, creating future opportunities for in vivo mechanistic studies.

We present evidence that p53 oligomers and fibrils form in late Braak stage AD brain tissue, but not age-matched controls, using Western blot and conformation-specific antibodies that identify the unique conformation of oligomers and fibrils. Although this is the first report of p53 oligomers and fibrils in AD, Uberti et al. have reported a conformationally altered form of p53 in AD patients [14, 63, 111]. It is possible this conformationally altered p53 is indeed oligomeric. In the present study, we focused on oligomer formation since this soluble intermediary species of aggregates is reported to be the most toxic species in AD [9, 25, 42, 59, 64, 65, 77, 99, 106], can seed/cross-seed the misfolding of other proteins, and therefore can affect the spread and severity of pathology [10, 53, 78, 107]. p53 is an inherently unstructured protein, making it prone to aggregate [8, 27], but this trait is also important to its functions, regulated by PTMs [89, 112]. Therefore, we hypothesized that PTMs may change the structure of p53 so that it may not succumb to oligomerization. However, phosphorylated p53 also oligomerized in AD brain. This suggests PTMs, and thereby changes in p53 structure, may not defend against seeding or aggregation. Furthermore, the seeding property of oligomers also seems to apply to p53 oligomers through our in vitro studies in Fig. 5. We also observed p53 and P-p53 along the microtubules in areas far away from the nucleus in untreated primary neurons, which suggests p53 can normally travel from one end of a neuron to another or possibly even from neuron to neuron. Indeed, p53 has been previously reported to be associated with microtubules [39] and found in synaptic terminals [40]. p53O were also observed along the microtubules far away from the nucleus (Fig. 5), which may be evidence of spreading. p53 aggregates have been previously reported to spread in a prion-like manner [4, 13, 34, 71, 93], however, additional experiments are needed to be conclusive for AD.

Our data demonstrates peri-nuclear distribution of p53O is consistent in late Braak stage human AD, aged htau mice, and in WT primary neurons exogenously treated with full length recombinant human p53O. Furthermore, this peri-nuclear distribution is also consistent with p53 aggregates in cancer [4, 66, 116, 120], which may indicate that the peri-nuclear distribution of p53 aggregates is a common factor across species and diseases. As a transcription factor, p53 serves its function in the nucleus, however, aggregation may inhibit nuclear import and thereby cause loss of function. Although we used late Braak stage (stage 6) human brain, a previous report by Silva et al. found nuclear p53 levels are highest in normal aging brains, but significantly reduced in pathological human AD brain, and reduced even more so in clinical-pathological AD brain [103]. This would suggest that a loss of nuclear, functional p53 is associated with the progression of pathology in AD. This loss of nuclear p53, which is consistent with our findings, may be due to its aggregation. p53 has been reported to be sequestered to the cytoplasm when aggregated, causing loss of function in neuroblastomas [81, 82, 117, 118]. Also, even if p53O were able to move into the nucleus, Lasagna-Reeves et al. demonstrated that aggregated p53 cannot bind to DNA [66], further supporting a loss of p53 function following aggregation.

### p53-mediated DNA damage response is impaired in AD

As p53 is a major signaling hub for assessing DNA damage and orchestrating a response, we investigated if p53 aggregation caused a loss of function in p53-mediated DNA damage response in AD brain. Indeed, multiple p53-mediated DNA DSB damage responders were reduced and/or mislocalized in AD despite evidence of significant DNA DSB damage. From this we conclude that p53 cannot orchestrate a robust response to DNA damage in AD, which may be due to p53 aggregation and/or interactions with tauO. Furthermore, we showed that P-p53, a PTM specific to DNA damage, forms oligomers in human AD and colocalizes with tauO in primary neurons. In both cases, P-p53 was mislocalized in the cytoplasm even in the presence of significant DNA damage. Together, this suggests p53 aggregation causes a loss of nuclear p53 function in the p53-mediated DNA damage response in human AD. This may cause significant genome instability to both mitochondrial and nuclear DNA and initiate signals for cell death. However, if p53 nuclear function is inhibited, controlled cell death through apoptosis may not be feasible, thereby causing the long neurodegenerative process seen in AD.

### p53 interacts with tau oligomers in Alzheimer’s disease

Tau is a major contributor to pathology in AD. Therefore, we wanted to assess whether tau may influence p53 function or aggregation. It has been reported that p53 under normal conditions, associates with microtubules in vitro [39]. This normal, close physical association of p53 with microtubules may explain the small colocalized region of p53 and WT tau observed in the human control (Fig. 3D2, G). This inherent proximity of p53 to tau increases the probability of this normal interaction being corrupted through pathologic tau. Although we found evidence that p53 and tau interact in AD, we postulate that a large proportion of the total tau observed in Fig. 3a, e colocalized with p53, was indeed oligomeric since tauO strongly colocalized with p53 in the AD tissue in the same pattern. p53 has been reported in neurites near Aβ plaques and in tau neuropils, but not NFTs in AD brain [28]. We also did not observe p53 with hyperphosphorylated NFTs, and minimal association with AT8 positive aggregates in human AD (data not shown). However the relationship between tau PTMs and oligomerization, as well as the phosphorylation sites which may trigger tau oligomerization, are still unknown [109]. Together, this suggests p53 may act at an earlier stage of pathology when tau oligomers initially arise. Although amyloid beta was not investigated in this study, future investigations of amyloid beta plaques and oligomers in relation to p53 aggregation may also help ascertain how p53 aggregates fit in the context of whole protein deposition and the timeline of disease pathology expected to occur in AD [21, 84].

Evidence presented here suggests the interaction between p53 and tau could be mediated by protein misfolding and/or the context of the disease. For example, in p53, the DNA binding domain, N-terminal domain, and C-terminal basic domains are all intrinsically disordered [27, 70] and prone to misfold. Tau is also intrinsically disordered [112] and therefore has many aggregation prone segments that could interact with p53. In contrast, many facets of AD pathology including impaired proteostasis, DNA damage, inflammation and mitochondrial dysfunction could also facilitate aggregation and interaction of p53 and tau [86, 95]. At this stage, we cannot exclude either. Future studies could investigate the specific domains or amino acids responsible for facilitating protein-protein interactions between p53 and tau/tauO in neurodegeneration [26].

It has also been reported that disruption of normal microtubule dynamics impedes p53 nuclear translocation and, in turn, inhibits activation of downstream targets by p53 [39], thereby demonstrating that an intact microtubule network is necessary for normal p53 trafficking. Destabilization of the microtubule network in AD, caused by the disruption of normal tau function, can cause disruption in cytoplasmic-nuclear transport [29, 91, 115]. Therefore, in addition to aggregation, disruption of microtubule dynamics may further explain the mislocalization of p53 and P-p53 outside the nucleus in human AD. Pathological forms of tau have also been shown to disrupt the nuclear pore [29] and nuclear membrane [91], which may impede nuclear accumulation of p53, causing it to instead accumulate outside the nucleus, which we observed in both AD brain and tau overexpressing mice.

Both tau and p53 have been independently reported to cross-seed, but never with each other. Our data indicates that tau cross-seeds p53, which was supported by our human data and primary neuron treatments with tauO. However, longer timepoints with a higher concentration of p53O may show different results. In addition, the fact that tauO colocalized with p53 in just 1 h would suggest that this is a relatively fast interaction and p53 loss of function occurs quickly. This observation extends to P-p53 as well. It was previously reported that tauO interaction with other proteins leads to greater toxicity than tauO alone [15, 85]. TauO interaction with p53 may have a similar outcome. However, given that WT primary neurons treated with p53O also showed mislocalization outside the nucleus suggests that tauO alone may not be the only contributor to p53 mislocalization.

TauO may also affect p53 through DNA damage as numerous publications have shown a link between tau and DNA damage [35, 108]. Pathological tau has been reported to cause heterochromatin relaxation while WT tau has been reported to protect DNA [35, 108]. Pathological tau may increase the stress in the cell, which could cause more p53 to move to the nucleus, setting up an opportunity for tauO to interact, cross-seed, or otherwise disrupt p53. Of note, the orthogonal view in AD brain tissue from Fig. 3k showed evidence of tauO in the nucleus, which could be contributing to the increase in DNA damage observed or causing unknown pathology. In addition, a recent report by Sola et al. showed that acute DNA damage combined with tau depletion caused altered p53 stability and activity, resulting in reduced cell death and increased cell senescence [105]. This would suggest that tau depletion negatively affects p53 function. Therefore, depletion of pathologic tau and/or tauO may restore p53 and the microtubules it requires for transport, better protecting cells from neurodegeneration.

**Fig. 6:**
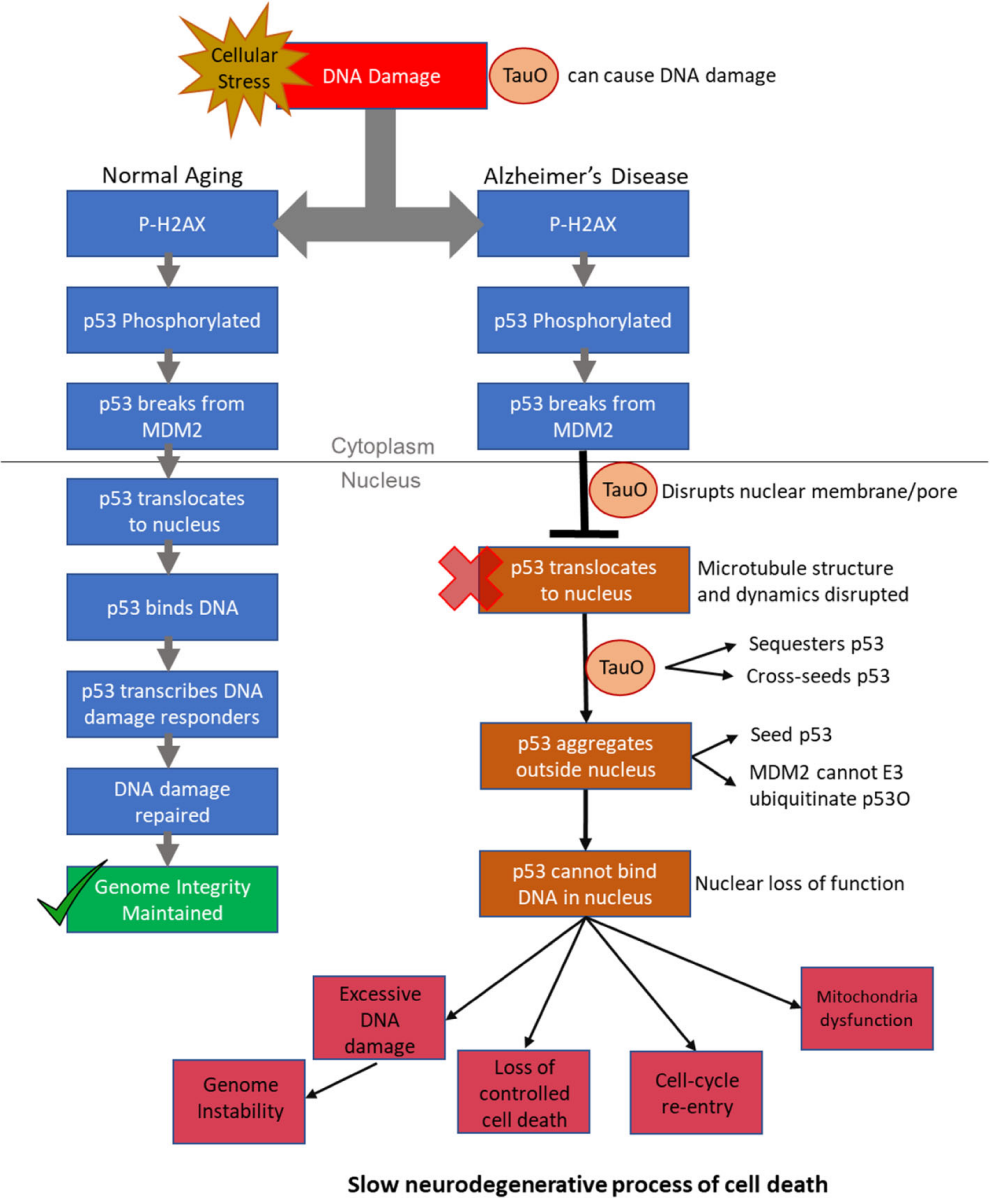
Proposed mechanism of p53 in Alzheimer’s disease pathology. (Left) Functional p53 response in normal aging where every day cellular stress causes production of DNA damage, p53 is altered by post-translational modifications to move to the nucleus and induce transcription of target genes to ameliorate damage. (Right) In AD disease pathology, the cell becomes stressed and activates p53 to address DNA damage and oxidative stress. However, due to breakdown of the microtubule network and tau oligomer pathology, p53 transport cannot enter the nucleus and accumulates outside the nucleus. Over time p53 may become unstable and start to aggregate. tauO near the nucleus interact with p53, causing sequestration and cross-seeding. P53 that cannot enter the nucleus will result in loss of nuclear function, causing dysfunction in critical cell function such as cell cycle arrest, DNA damage repair, and apoptosis. With no repair nor a way to perform controlled cell death, conditions in the cell will continue to deteriorate, promoting aggregation of other intrinsically disordered proteins

In summary, we present evidence that p53 can form aggregates, mislocalize, form interactions with pathological tauO, which together, may contribute to impairment in the p53-mediated DNA damage response in human AD. Our proposed mechanism for p53 contribution to AD pathology (Fig. 6): increased cellular stress and/or DNA damage in AD causes activation of p53. However, due to breakdown of the microtubule network and tau oligomer pathology, p53 cannot enter the nucleus and accumulates outside the nucleus. Over time, p53 may become unstable and start to aggregate, further inhibiting its access to the nucleus. tauO, near the nucleus, may at the same time interact with p53, cross-seeding p53, to form p53O. WT p53 that cannot enter the nucleus will result in loss of function, causing further dysfunction in critical cell functions such as cell cycle arrest, DNA damage repair, and apoptosis. With no repair, nor a way to perform controlled cell death, conditions in the cell will continue to deteriorate, promoting additional aggregation of other intrinsically disordered proteins that can form oligomers, cause toxicity, and spread disease.

## Conclusions

Alzheimer’s disease is a devastating neurodegenerative disease that affects millions worldwide and remains without effective diagnostics nor effective therapies. While the breadth of studies aimed at understanding the biological mechanisms surrounding protein aggregation in AD and other neurodegenerative diseases is continuously growing, much is left to be understood. Disruption of p53, through aggregation and interactions with tauO, needs to be further investigated in AD as it may provide novel cellular targets for future therapeutics. Our results indicate that targeting pathological tau, specifically tauO, may prevent aggregation and disruption of p53. How DNA damage and aggregation may be linked as either cause or consequence also needs to be further investigated. Indeed, as p53 controls many cell functions, affecting this one critical transcription factor may set an irreversible course towards AD. Our results also suggest that the role of p53 in AD and other tauopathies warrants additional investigation.

## Supplementary information

**Additional file 1: Table S1.** Human Sample Information from University of Kentucky Alzheimer’s Disease Center Brain Bank. Figure Legend: List of human Alzheimer’s disease and control brain tissue used in this study with information pertinent to neuropathology.

**Additional file 2: Figure S1.** Recombinant human p53 purification, SEC fractionation by FPLC and oligomer confirmation by AFM. (A) Representative image of different p53 purification elutions tested by western blot using anti-p53 antibody shows detection of p53 monomer (53 kD) and high molecular weight p53 formation. (B) High molecular weight p53 from purified recombinant p53 elutions are resistant to 8 M urea and boiling treatments by western blot. (C) Representative image of p53 fractions separated by Size Exclusion chromatography (SEC) on FPLC showing separation of p53 monomer (53 kD) from higher molecular weight bands by western blot. (D) p53 protein fractions show monomers and size and spherical shape consistent with p53 oligomers by AFM. (E) Graphical representation of size distribution of p53O from D. Scale bar = 100 nm.

**Additional file 3: Figure S2.** Alexa-Fluor labeled p53 oligomers and tau oligomers internalize to the nucleus of C57Bl/6 primary neurons. (A-B) Representative confocal images of C57Bl/6 primary neurons immunofluorescently probed with anti-β-III-Tubulin (green) and anti-p53 (red; only untreated). Magnified ROI from merged images demonstrates endogenous p53 and AFL-p53O are within the confines of β-III-Tubulin, suggesting internalization and localization near the nucleus. (C-D) Representative confocal images with same conditions as (A-B), but with tau. Magnified ROI from merged image demonstrates AFL-tauO within the confines of β-III-Tubulin, suggesting they are internalized by the cell and localize to the nucleus. (E, G) Representative confocal images with untreated and tauO treated neurons immunofluorescently probed with anti-tau (red; only untreated), and anti-P-H2AX. Magnified ROI show P-H2AX in the nucleus (F, H) with significantly more (G) P-H2AX fluorescent intensity signal in tauO treated neurons. Keyence Microscope. Scale bar =50 μm.

**Additional file 4: Figure S3.** Toxicity of p53 monomer, oligomer, fibril, and mixtures in primary neurons by LDH Assay. (A) C57BL/6 primary neurons (*n* = 2) treated with 0.5 μM and (B) 1 μM p53 monomer, p53 oligomer, p53 fibril, and p53 mixtures (each treatment performed in triplicate) show no toxicity by LDH assay (C) Tau KO primary neurons (*n* = 1) treated with 0.5 μM and (D) 1 μM p53 monomer, p53 oligomer, p53 fibril, and p53 mixtures (each treatment performed in triplicate) show no toxicity by LDH assay.

## Data Availability

The datasets used and/or analysed during the current study available from the corresponding author on reasonable request.

## Abbreviations

AD: Alzheimer’s disease
AFL-p53O: Alexa-Fluor labeled p53 oligomer
AFL-tauO: Alexa-Fluor labeled tau oligomer
Ctrl: Control
DSBs: Double-strand breaks
FPLC: Fast Protein Liquid Chromatography
KO: Knockout
NFT: Neurofibrillary tangles
P301L mice: Tg2576/Tau (P301L) transgenic mice
p53O: P53 oligomer
PBS: Phosphate buffered saline
PCC: Pearson’s Correlation coefficient
P-H2AX: Phosphorylated histone H2AX
PLA: Proximity ligation assay
P-p53: Phosphorylated p53
PTMs: Post-translational modifications
ROI: Region of interest
rp53: Recombinant human p53
SEC: Size-exclusion chromatography
tauO: Tau oligomer
WT: Wild type
