# Supplementary material for: P53 aggregation, interactions with tau, and impaired DNA damage response in Alzheimer’s disease
Source: Acta Neuropathol Commun. 2020 Aug 10;8:132. doi: 10.1186/s40478-020-01012-6 (PMC7418370; doi:10.1186/s40478-020-01012-6)
Supplement: Supplementary file 1 — Additional file 1: Table S1. Human Sample Information from University of Kentucky Alzheimer’s Disease Center Brain Bank. Figure Legend: List of human Alzheimer’s disease and control brain tissue used in this study with information pertinent to neuropathology. [file 40478_2020_1012_MOESM1_ESM.docx]

**Supplemental Table 1: Human Sample Information from University of Kentucky Alzheimer’s Disease Center Brain Bank**

| **Human Sample Information from University of Kentucky Brain Bank** | | | | | |
| --- | --- | --- | --- | --- | --- |
| **Sample ID** | **Neuropath** | **Age** | **Sex** | **PMI** | **Braak Stage** |
| 1076 | AD | 70 | F | 3.25 | 6 |
| 1086 | AD | 90 | F | 2.75 | 6 |
| 1098 | AD | 81 | F | 2.75 | 5 |
| 1143 | AD | 78 | M | 3.5 | 6 |
| 1188 | AD | 75 | F | 2.5 | 6 |
| 1120 | AD | 83 | F | 4.75 | 6 |
| 1104 | AD | 90 | M | 3.25 | 6 |
| 1154 | AD | 86 | M | 3.25 | 6 |
| 1199 | AD | 88 | M | 2.75 | 6 |
| 1092 | Control | 86 | F | 1.75 | 1 |
| 1103 | Control | 76 | F | 2 | 1 |
| 1216 | Control | 79 | F | 1.75 | 1 |
| 1244 | Control | 90 | F | 2.25 | 2 |
| 1271 | Control | 71 | M | 2.6 | 0 |
| 1161 | Control | 84 | F | 2.5 | 0 |
| 5091 | Control | 81 | M | 12 | 1 |
| 1170 | Control | 84 | F | 2.5 | 1 |
| 1131 | Control | 80 | F | 2.25 | 1 |

Figure Legend: List of human Alzheimer’s disease and control brain tissue used in this study with information pertinent to neuropathology.
