# Supplementary material for: P53 aggregation, interactions with tau, and impaired DNA damage response in Alzheimer’s disease
Source: Acta Neuropathol Commun. 2020 Aug 10;8:132. doi: 10.1186/s40478-020-01012-6 (PMC7418370; doi:10.1186/s40478-020-01012-6)
Supplement: Supplementary file 2 — Additional file 2: Figure S1. Recombinant human p53 purification, SEC fractionation by FPLC and oligomer confirmation by AFM. (A) Representative image of different p53 purification elutions tested by western blot using anti-p53 antibody shows detection of p53 monomer (53 kD) and high molecular weight p53 formation. (B) High molecular weight p53 from purified recombinant p53 elutions are resistant to 8 M urea and boiling treatments by western blot. (C) Representative image of p53 fractions separated by Size Exclusion chromatography (SEC) on FPLC showing separation of p53 monomer (53 kD) from higher molecular weight bands by western blot. (D) p53 protein fractions show monomers and size and spherical shape consistent with p53 oligomers by AFM. (E) Graphical representation of size distribution of p53O from D. Scale bar = 100 nm. [file 40478_2020_1012_MOESM2_ESM.pptx]

## Slide 1
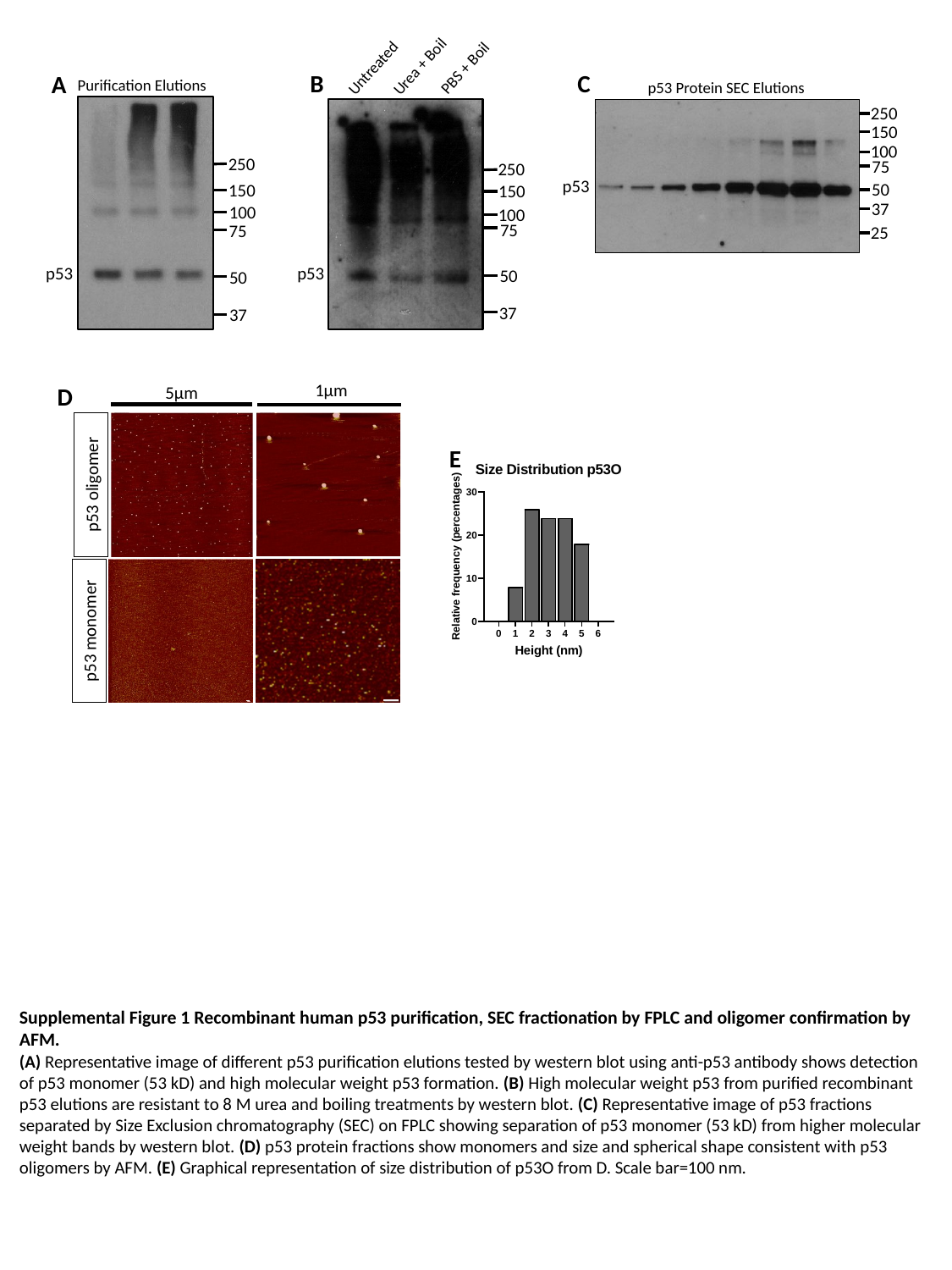

Urea + Boil
PBS + Boil
Untreated
B
250
150
100
75
p53
50
37
C
250
150
100
75
p53
50
37
25
A
250
150
100
75
p53
50
37
Purification Elutions
p53 Protein SEC Elutions
1µm
5µm
D
E
p53 oligomer
p53 monomer
Supplemental Figure 1 Recombinant human p53 purification, SEC fractionation by FPLC and oligomer confirmation by AFM.
(A) Representative image of different p53 purification elutions tested by western blot using anti-p53 antibody shows detection of p53 monomer (53 kD) and high molecular weight p53 formation. (B) High molecular weight p53 from purified recombinant p53 elutions are resistant to 8 M urea and boiling treatments by western blot. (C) Representative image of p53 fractions separated by Size Exclusion chromatography (SEC) on FPLC showing separation of p53 monomer (53 kD) from higher molecular weight bands by western blot. (D) p53 protein fractions show monomers and size and spherical shape consistent with p53 oligomers by AFM. (E) Graphical representation of size distribution of p53O from D. Scale bar=100 nm.
