# Supplementary material for: P53 aggregation, interactions with tau, and impaired DNA damage response in Alzheimer’s disease
Source: Acta Neuropathol Commun. 2020 Aug 10;8:132. doi: 10.1186/s40478-020-01012-6 (PMC7418370; doi:10.1186/s40478-020-01012-6)
Supplement: Supplementary file 3 — Additional file 3: Figure S2. Alexa-Fluor labeled p53 oligomers and tau oligomers internalize to the nucleus of C57Bl/6 primary neurons. (A-B) Representative confocal images of C57Bl/6 primary neurons immunofluorescently probed with anti-β-III-Tubulin (green) and anti-p53 (red; only untreated). Magnified ROI from merged images demonstrates endogenous p53 and AFL-p53O are within the confines of β-III-Tubulin, suggesting internalization and localization near the nucleus. (C-D) Representative confocal images with same conditions as (A-B), but with tau. Magnified ROI from merged image demonstrates AFL-tauO within the confines of β-III-Tubulin, suggesting they are internalized by the cell and localize to the nucleus. (E, G) Representative confocal images with untreated and tauO treated neurons immunofluorescently probed with anti-tau (red; only untreated), and anti-P-H2AX. Magnified ROI show P-H2AX in the nucleus (F, H) with significantly more (G) P-H2AX fluorescent intensity signal in tauO treated neurons. Keyence Microscope. Scale bar =50 μm. [file 40478_2020_1012_MOESM3_ESM.pptx]

## Slide 1
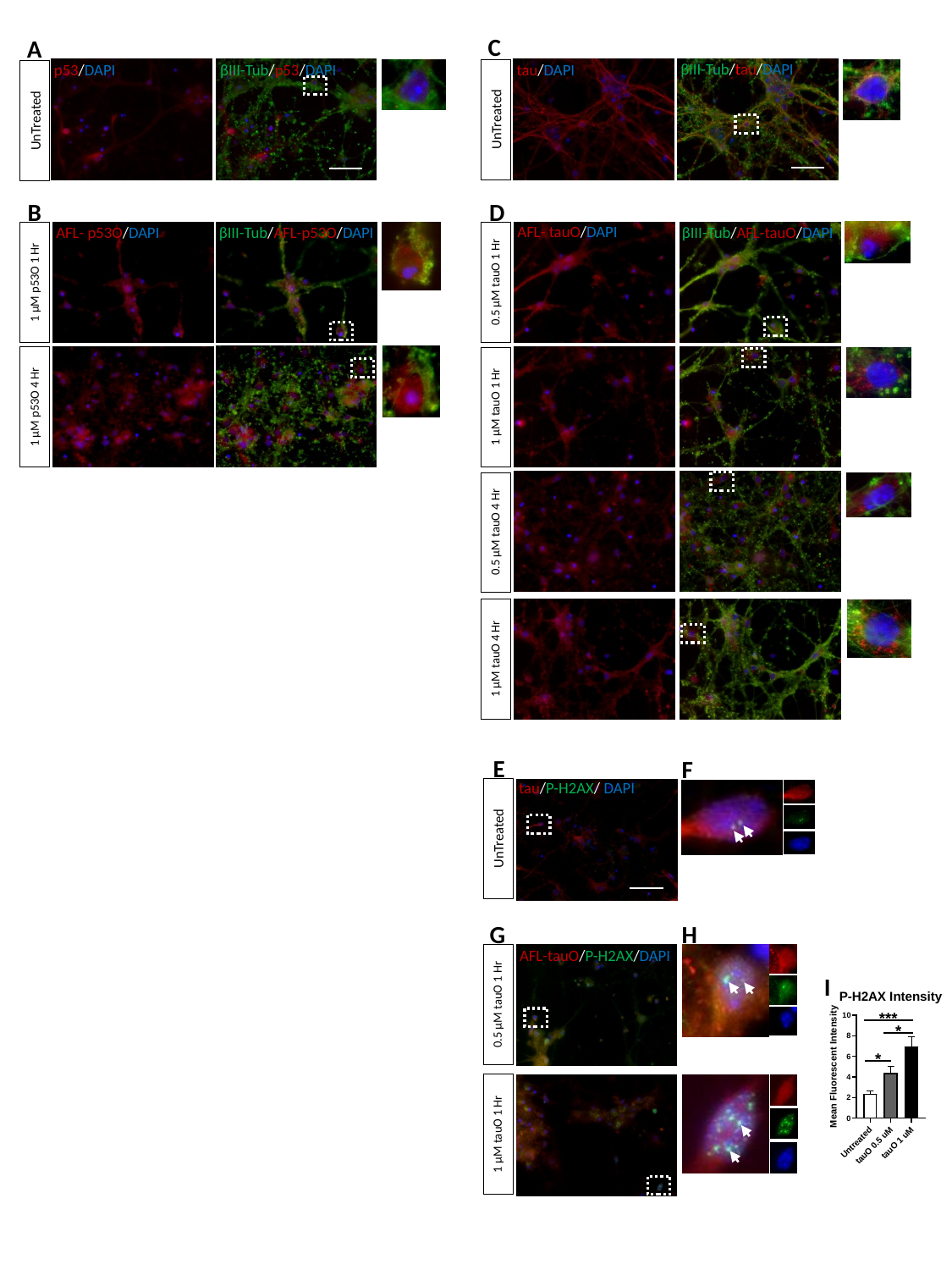

C
A
βIII-Tub/tau/DAPI
p53/DAPI
tau/DAPI
βIII-Tub/p53/DAPI
UnTreated
UnTreated
D
B
AFL- tauO/DAPI
βIII-Tub/AFL-p53O/DAPI
AFL- p53O/DAPI
βIII-Tub/AFL-tauO/DAPI
1 μM p53O 1 Hr
0.5 μM tauO 1 Hr
1 μM p53O 4 Hr
1 μM tauO 1 Hr
0.5 μM tauO 4 Hr
1 μM tauO 4 Hr
E
F
tau/P-H2AX/ DAPI
UnTreated
G
H
AFL-tauO/P-H2AX/DAPI
I
0.5 μM tauO 1 Hr
1 μM tauO 1 Hr

## Slide 2
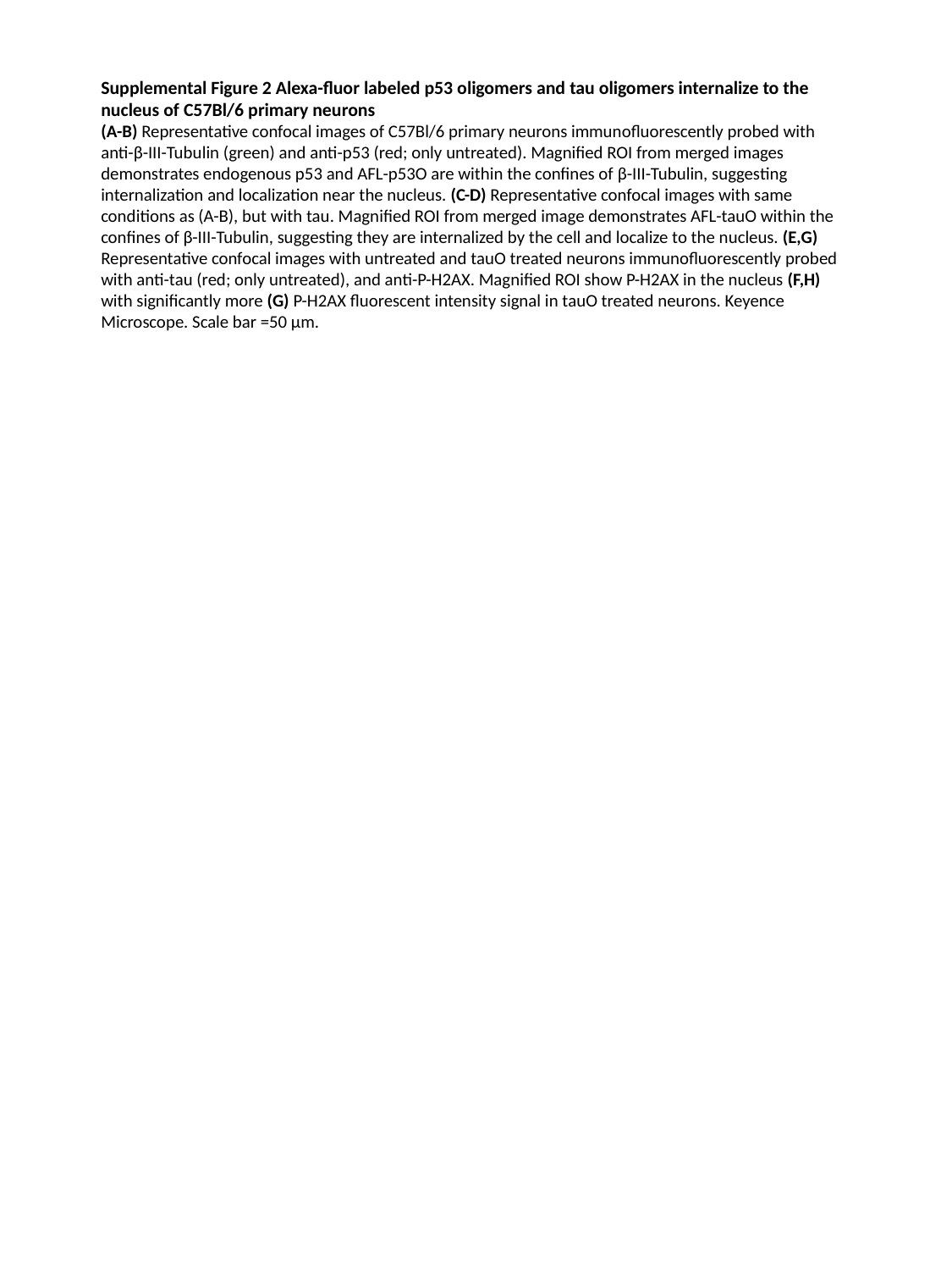

Supplemental Figure 2 Alexa-fluor labeled p53 oligomers and tau oligomers internalize to the nucleus of C57Bl/6 primary neurons
(A-B) Representative confocal images of C57Bl/6 primary neurons immunofluorescently probed with anti-β-III-Tubulin (green) and anti-p53 (red; only untreated). Magnified ROI from merged images demonstrates endogenous p53 and AFL-p53O are within the confines of β-III-Tubulin, suggesting internalization and localization near the nucleus. (C-D) Representative confocal images with same conditions as (A-B), but with tau. Magnified ROI from merged image demonstrates AFL-tauO within the confines of β-III-Tubulin, suggesting they are internalized by the cell and localize to the nucleus. (E,G) Representative confocal images with untreated and tauO treated neurons immunofluorescently probed with anti-tau (red; only untreated), and anti-P-H2AX. Magnified ROI show P-H2AX in the nucleus (F,H) with significantly more (G) P-H2AX fluorescent intensity signal in tauO treated neurons. Keyence Microscope. Scale bar =50 µm.
