# Supplementary material for: P53 aggregation, interactions with tau, and impaired DNA damage response in Alzheimer’s disease
Source: Acta Neuropathol Commun. 2020 Aug 10;8:132. doi: 10.1186/s40478-020-01012-6 (PMC7418370; doi:10.1186/s40478-020-01012-6)
Supplement: Supplementary file 4 — Additional file 4: Figure S3. Toxicity of p53 monomer, oligomer, fibril, and mixtures in primary neurons by LDH Assay. (A) C57BL/6 primary neurons (n = 2) treated with 0.5 μM and (B) 1 μM p53 monomer, p53 oligomer, p53 fibril, and p53 mixtures (each treatment performed in triplicate) show no toxicity by LDH assay (C) Tau KO primary neurons (n = 1) treated with 0.5 μM and (D) 1 μM p53 monomer, p53 oligomer, p53 fibril, and p53 mixtures (each treatment performed in triplicate) show no toxicity by LDH assay. [file 40478_2020_1012_MOESM4_ESM.pptx]

## Slide 1
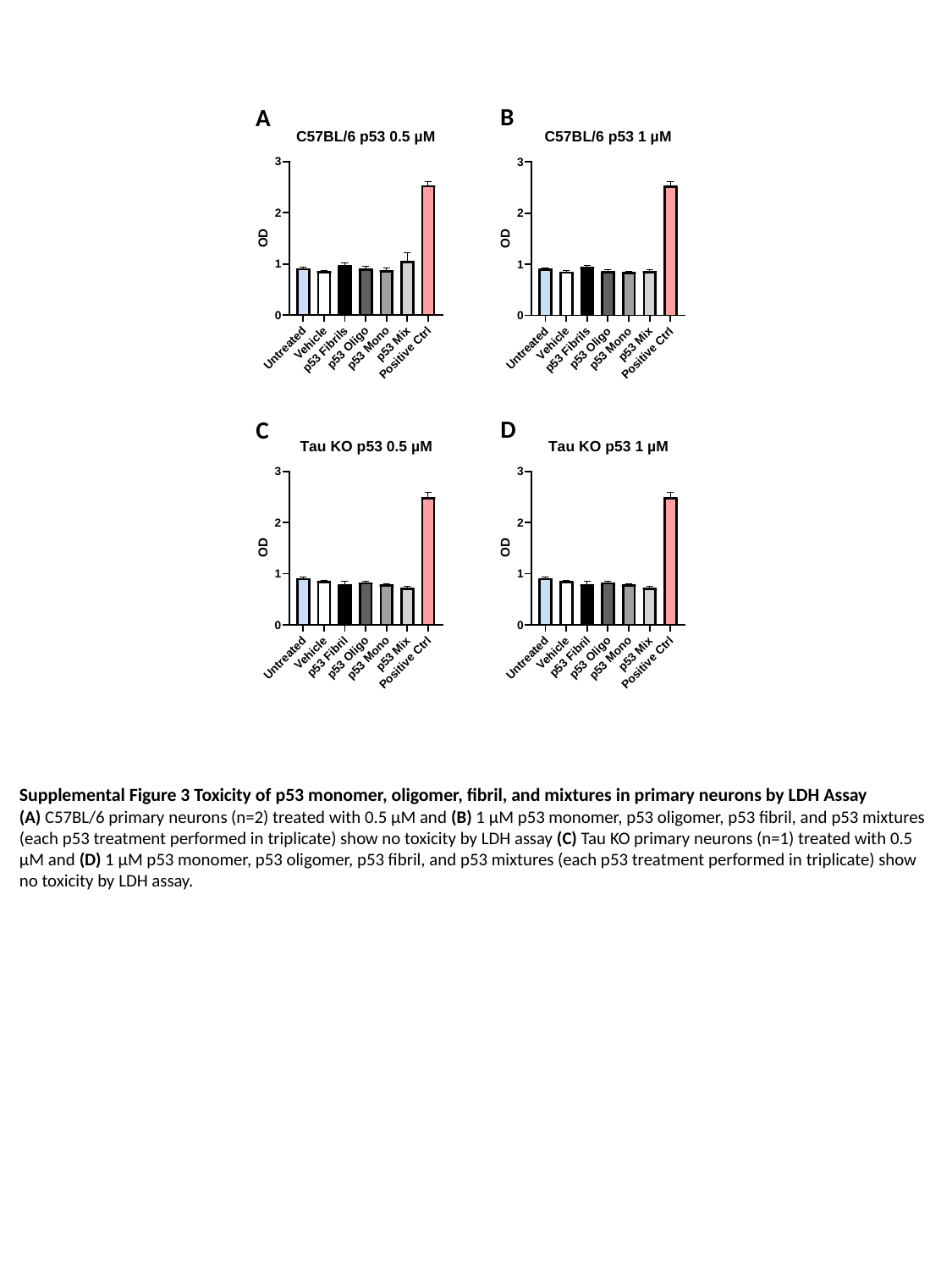

B
A
D
C
Supplemental Figure 3 Toxicity of p53 monomer, oligomer, fibril, and mixtures in primary neurons by LDH Assay
(A) C57BL/6 primary neurons (n=2) treated with 0.5 µM and (B) 1 µM p53 monomer, p53 oligomer, p53 fibril, and p53 mixtures (each p53 treatment performed in triplicate) show no toxicity by LDH assay (C) Tau KO primary neurons (n=1) treated with 0.5 µM and (D) 1 µM p53 monomer, p53 oligomer, p53 fibril, and p53 mixtures (each p53 treatment performed in triplicate) show no toxicity by LDH assay.
